# The relationship between online learning self-efficacy, informal digital learning of English, and student engagement in online classes: the mediating role of social presence

**DOI:** 10.3389/fpsyg.2023.1266009

**Published:** 2023-10-02

**Authors:** Ronglan Wu

**Affiliations:** School of Humanity and Foreign Languages, Zhejiang Shuren University, Hangzhou, China

**Keywords:** online learning self-efficacy, informal digital learning of English, student engagement, online classes, social presence, EFL students

## Abstract

**Introduction:**

This study investigates the interplay between Online Learning Self-Efficacy (OLSE), Informal Digital Learning of English (IDLE), student engagement in online classes, and the mediating effect of Social Presence (SP) among intermediate Chinese English as a Foreign Language (EFL) students. The research sample consisted of 389 participants enrolled in online English courses within a chain of language schools.

**Methods:**

Data collection involved the use of validated scales to assess OLSE, IDLE, SP, and online student engagement. Structural Equation Modeling (SEM) was employed for data analysis.

**Results:**

The findings revealed direct influences of OLSE and SP on online student engagement. Additionally, IDLE exhibited a positive impact on online student engagement, with a partial mediation effect through SP. This suggests that informal digital English learning significantly contributes to students’ engagement in online classes, with this influence being facilitated by the sense of social presence experienced by students during virtual interactions.

**Discussion:**

This research underscores the importance of OLSE, IDLE, and SP in shaping student engagement within online learning environments. The results highlight that fostering informal digital English learning can enhance students’ active participation in online courses, with SP serving as a key mediator in this relationship. These insights provide valuable guidance for educators and institutions seeking to improve student engagement in online educational settings.

## Introduction

In recent years, the rapid advancement of digital technologies has transformed the landscape of education, ushering in a new era of online learning. This paradigm shift has been particularly pronounced in English as a Foreign Language (EFL) education, where online learning has gained prominence (Abdelrady and Akram, 2022; Fathi and Rahimi, 2022; Hasibuan et al., 2022; Jiang et al., 2023). Online education, with its potential to offer high-quality learning experiences, has significantly enriched the Second Language Acquisition (SLA) process (Hwang et al., 2016; Tai, 2016; Lei et al., 2022). However, within this digital realm, a critical challenge arises: how to foster active student participation and engagement. Student engagement, reflecting the extent of students’ involvement in educational activities leading to desired learning outcomes, is a linchpin in ensuring the effectiveness of online learning (Peng, 2017; Lei et al., 2018).

The concept of student engagement takes on a multifaceted dimension in online courses, where challenges like indirect social interactions, suboptimal student-teacher and peer relationships, and the need for learners to adjust to the virtual setting are prevalent (Derakhshesh et al., 2022; Yuyun, 2023). Furthermore, maintaining consistent and meaningful student involvement can be challenging, as issues related to persistence and efficiency often surface (Lorenzo, 2012). Rahman (2021) highlighted that insufficient student engagement stands as a major hurdle in online learning. Consequently, there’s a compelling need to delve deeper into the factors that drive students’ online learning engagement, particularly within the context of SLA (Dixson, 2012, 2015; Gamage et al., 2022; Salas-Pilco et al., 2022). A nuanced understanding of student engagement can provide insights into learners’ active participation and academic achievements in language learning, thus informing better language teaching practices.

One such influential factor that appears to significantly impact student engagement is Informal Digital Learning of English (IDLE). This emerging phenomenon in EFL classrooms encompasses self-directed language tasks carried out in informal digital settings, often driven by learners’ personal interests and curiosity, and independent of direct teacher guidance (Lee and Dressman, 2018; Lee, 2019, 2020; Soyoof et al., 2023). Despite the growing attention IDLE has garnered in language learning contexts, its correlation with student engagement remains largely unexplored in the realm of second language (L2) education. Consequently, there’s a pressing need to investigate this variable, especially concerning its impact on the online learning engagement of EFL learners. Such an exploration could uncover valuable insights into the potential role of IDLE in enhancing learners’ commitment and active participation in virtual language learning experiences.

Notably, while student engagement has been studied extensively in other educational settings (Kuh, 2009; Lee, 2014), it remains an underexplored area in L2 contexts (Hoomanfard et al., 2021). Furthermore, the specific factors influencing online learning engagement among EFL students have received limited attention, despite the increasing importance of this issue (Schaeffer and Konetes, 2010; El-Sayad et al., 2021). To address this gap, our study set out to explore how online engagement is influenced by online learning self-efficacy, social presence, and IDLE among Chinese EFL students. By examining these relationships within the virtual context, our research aims to contribute to the existing literature and provide valuable insights into the interplay of these factors, ultimately enhancing EFL learners’ engagement in online learning.

Theoretically, our study is grounded in Bandura’s (1977) social cognitive theory, which suggests that individuals’ self-efficacy beliefs, such as online learning self-efficacy (OLSE), play a central role in influencing their engagement and behavior in a given context. In the context of online education, self-efficacy beliefs can significantly impact students’ motivation, persistence, and willingness to engage with course materials and peers (Bandura, 2006; Chang et al., 2014). Therefore, it is theoretically plausible to posit that OLSE, as a measure of students’ confidence in their ability to succeed in online learning, should relate positively to their online engagement.

Moreover, the concept of social presence, rooted in Mehrabian’s (1969) idea of immediacy, is theoretically linked to students’ engagement in online classes. Social presence represents the extent to which individuals project their authentic selves and engage in meaningful interactions in a mediated environment (Garrison, 2007). Higher levels of social presence are expected to foster a sense of comfort and satisfaction in online interactions with teachers and peers, ultimately contributing to student engagement (Aragon, 2003). Thus, there is a conceptual basis for hypothesizing a positive relationship between social presence and student engagement in online classes.

Lastly, the role of IDLE in shaping student engagement can be theoretically linked to the idea of extending language learning beyond the formal classroom. IDLE represents self-directed language learning activities in informal digital settings, which provide learners with additional exposure to the language and opportunities for practice (Lee and Xie, 2023). This extended language exposure and practice may naturally lead to heightened language proficiency, which, in turn, can contribute to increased student engagement in online classes. Therefore, there is a theoretical rationale for investigating the relationship between IDLE and student engagement in the online learning context.

Overall, our research is guided by well-established theories and conceptual frameworks, suggesting that OLSE, social presence, and IDLE are theoretically and conceptually linked to student engagement in online classes. By empirically examining these relationships, our study aims to provide a deeper understanding of the factors that influence EFL learners’ online engagement, ultimately contributing to the enhancement of online language learning experiences.

## Literature review

### Online learning engagement

Engagement, conceptualized as a multi-faceted construct (Jimerson et al., 2003), refers to the depth of productive participation and persistence in an activity (Ben-Eliyahu et al., 2018). In the educational context, engagement encompasses aspirations, belonging, and productivity (Hazel et al., 2013). Specifically, student engagement is commonly understood as learners’ eagerness, motivation, and drive to actively participate and achieve success in their own learning (Zepke and Leach, 2010; Zhang and Hyland, 2022).

Over time, the conceptualization of student engagement has evolved from a single-dimensional approach to a multi-dimensional construct. Initially, researchers primarily focused on the behavioral dimension, which includes positive attitudes and learning behaviors while excluding negative experiences during learning activities (Engels et al., 2016). Subsequently, they explored the behavioral and emotional dimensions, and finally, the cognitive dimension (Hu and Li, 2017). As a result, student engagement is now understood to consist of four dimensions: behavioral, emotional, cognitive, and social engagement (Fredricks et al., 2004; Wang et al., 2016; Mohammad Hosseini et al., 2022).

Behavioral engagement involves positive attitudes and behaviors toward learning activities, excluding negative behaviors (Engels et al., 2016). Emotional engagement encompasses students’ positive affective and emotional reactions toward teachers, classmates, and academic content during learning activities (da Rocha Seixas et al., 2016; Liu et al., 2022). Cognitive engagement emphasizes the importance of positive self-regulation, learning strategies, and cognitive efforts to excel in specific learning contexts (Huang et al., 2022). Lastly, social engagement refers to students’ inclination to interact with instructors, classmates, the subject matter, and maintain interpersonal communication (Johnston, 2018; Liu et al., 2023). Each of these dimensions possesses unique qualities and significantly influences students’ level of engagement in educational settings (Derakhshan et al., 2022; Yu et al., 2022). Understanding the multi-dimensional nature of student engagement is crucial for educators to foster a more enriching and supportive learning environment that promotes active and committed participation among students.

After the COVID-19 pandemic, almost all countries incorporated digital tools and this has led to a sharp increase in learning classrooms. This shift toward online systems has its own critical challenges for L2 students and teachers. Specifically, student engagement in online EFL classrooms is of high importance as learners are distant from their peers and instructors (Akbari et al., 2016). In the realm of EFL, online engagement is related to learners being actively ad effectively engaged in virtual classes by reflecting on the content and sharing ideas and interacting with peers and teachers.

Previous research has tried to investigate the potential antecedent of online learning engagement among students (e.g., Ferrer et al., 2020; Chen et al., 2022). For instance, Chen et al. (2022) tended to address the role of a student-facing social learning analytics tool in impacting student engagement in online collaborative writing. Employing a mixed-method design, their findings revealed that the student-facing social learning analytics tool exerted a strong influence on online engagement of learners. Drawing on self-determination theory, Ferrer et al. (2020) investigated the role of attitudes toward learning in affecting online learning engagement of students. Analyzing the data from 574 learners at an Australian higher education institution, the authors found that on students’ attitudes to online learning could have a great impact on learners’ online engagement.

As the above review of the existing literature indicates, the empirical evidence on online engagement has explored various sources contributed to this phenomenon. However, the association of learners’ perceptions, assurance, and anticipations with online engagement as well as the potential roles of other L2-related in shaping online engagement have remained unexamined in L2 learning and teaching.

### Online learning self-efficacy

In the context of the burgeoning online instructional landscape, L2 researchers have devoted attention to investigating the potential role of self-efficacy in shaping learners’ academic achievement within online language learning (Hsu et al., 2022). Rooted in the social cognitive theory (Bandura, 1977) and locus of control theory (Rotter, 1966), self-efficacy refers to individuals’ judgments of their capabilities to organize and execute actions necessary to accomplish specific performances (Bandura, 1986, p. 391). In simpler terms, self-efficacy pertains to individuals’ self-assessment of their abilities to successfully perform behaviors required for a particular task. This construct significantly contributes to learners’ competence, as individuals are more likely to achieve particular performance outcomes when they have confidence in their ability to handle and control the task (Bandura, 2006; Chang et al., 2014; Bernacki et al., 2015).

In the domain of SLA, self-efficacy holds a critical role as a central factor influencing L2 learners’ performance motivation and academic achievement (Namaziandost and Çakmak, 2020). According to Derakhshesh et al. (2022), self-efficacy significantly contributes to EFL students’ cognitive, affective, and physiological resources, supporting their pursuit of learning goals. It is well-established that L2 students with a strong sense of self-efficacy demonstrate increased perseverance in the face of challenges and exhibit greater resilience in coping with difficulties (Li, 2022; Rayyan et al., 2023). The extension of self-efficacy into the realm of SLA research has provided valuable insights into the impact of learners’ belief in their abilities on their language learning achievements, particularly in the dynamic online learning environment. Scholars continue to explore the multifaceted interplay between self-efficacy, academic performance, and motivation, contributing to a deeper understanding of the factors that foster successful language learning outcomes in the digital era.

The proliferation of computer-based technologies has paralleled the rise in the use of web and internet technologies, prompting researchers to investigate context-specific self-efficacy in virtual learning environments. Consequently, unique self-efficacy constructs specific to online contexts, such as computer self-efficacy, Internet self-efficacy, and online learning self-efficacy, have emerged as areas of interest (Gautam et al., 2020; Kuo et al., 2021). Specifically, online self-efficacy has been linked to other constructs, including online engagement, in previous research (e.g., Han et al., 2021; Heo et al., 2021; Kuo et al., 2021). For instance, in a study conducted by Kuo et al. (2021), the correlation between web-based (i.e., online) learning self-efficacy (i.e., OLSE) and online learning engagement was explored. Through data analysis from a sample of 4,285 students, the researchers discovered a positive impact of online learning self-efficacy on learners’ engagement in online courses. Similarly, Alemayehu and Chen (2021) explored the impact of online learning self-efficacy on online learning engagement among 354 students. Through structural equation modeling analysis, they demonstrated a significant positive effect of learning self-efficacy on engagement in the online environment.

In the EFL domain, only one study has been identified that examines the togetherness between L2 learners’ online self-efficacy and online engagement. Derakhshan and Fathi (2023) explored the interactive effect of online self-efficacy in predicting online engagement. Their findings indicated a positive influence of online learning self-efficacy on online learning engagement among EFL students. The investigation of self-efficacy constructs in the context of virtual learning environments is of paramount importance, as it provides valuable insights into the factors that can influence learners’ engagement and success in online educational settings. As researchers continue to delve into this area, a deeper understanding of the role of online self-efficacy in promoting engagement and achievement in virtual learning environments will undoubtedly contribute to the improvement and optimization of online language learning experiences.

Notwithstanding the fact that online self-efficacy is theoretically viewed as a critical factor for shaping learning engagement of L2 learners, there is little empirical evidence of its association with online engagement in the context of EFL learning and teaching. This being said, the current research contributes to that matter by investigating the potential role of online learning self-efficacy in affecting online learning engagement among EFL students.

### Social presence

Social presence, rooted in the concept of immediacy (Mehrabian, 1969), is a construct frequently studied in the literature of human-computer interactions and communications. In the context of online learning, researchers have sought to address learners’ feelings of isolation or loneliness in virtual classrooms, leading to the conceptualization of social presence as a means to enhance interpersonal connections (Lowenthal, 2010). Originally associated with conveying communication details, such as facial expressions and verbal intonation, social presence was initially considered a static attribute of the communication medium. However, over time, the understanding of social presence has evolved to encompass specific communications occurring in the medium and learners’ subjective perceptions of these communications (Wise et al., 2004; Wei et al., 2012).

As noted by Garrison (2007), social presence pertains to individuals’ capacity to project their authentic selves and engage in personal and purposeful interactions with others. Higher levels of social presence are thought to contribute to individuals’ comfort in social environments and their sense of satisfaction in interactions with teachers and peers (Aragon, 2003). Conceptualized as a multi-dimensional construct, social presence encompasses how much a student feels affectively connected to peers and to what extent they perceive themselves and others in the mediated context (Harvey et al., 2018; Whiteside et al., 2023). This multi-faceted approach to social presence enables a comprehensive understanding of the affective and communicative aspects that influence learners’ experiences in online learning environments.

In online environments, social presence can significantly contribute to learners’ academic performance, and increase the quality off learning process (Cui et al., 2013). It can help provide an environment in which students can perceive the learning process as comfortable, friendly and approachable, and by providing interesting, engaging, and intrinsically rewarding social interaction opportunities, it can help instigate and boost cognitive and affective learning goals (Aragon, 2003; Lim and Richardson, 2016). Recent studies have shown evidence that social presence can lead to engagement among students. In fact, it is argued that once learners who hold perceived social presence in virtual classrooms can experience a higher levels of learning satisfaction and engagement (Grieve et al., 2016). In an online environment, Molinillo et al. (2018) examined the impact of social presence on online engagement of learners. To this end, 416 students participated in the study. Their findings indicated that social presence had a positive influence on online engagement of students. Similarly, Doo and Bonk (2020) tended to facilitate student engagement by examining the impacts of self-efficacy, self-regulation and social presence on online learning engagement. Collecting data from 390 students, the results demonstrated that self-regulation, self-efficacy, and social presence positively shaped learners’ online learning engagement. Miao et al. (2022) examined the impact of social presence on learning engagement in online learning environments. Administering a survey to a sample of 354 students, their results confirmed the significant influence of social presence on online learning engagement among students.

Despite the limited number of previous studies in various educational settings, the investigation of social presence and its role in impacting online learning engagement in the context of EFL is rather non-existing. In this study, we tend to bridge this gap by examining the effect of social presence on learning engagement in online environment among EFL students.

### IDLE

Bax (2003) introduced the concept of “normalization” to envision the future of Computer-Assisted Language Learning (CALL), whereby CALL would seamlessly integrate into language learning processes, serving the needs of language learners and educators while becoming less overt in the learning environment. In recent times, as technological innovations have transformed education, including SLA, researchers and scholars have increasingly focused on the significance of L2 students’ informal language learning and usage in out-of-class and digital settings. This emerging field within CALL has delved into novel areas, particularly exploring the impact of technological tools on out-of-class autonomous language acquisition (Lee et al., 2023; Lee and Xie, 2023).

This shift in focus has given rise to a new concept called Informal Digital Learning of English (IDLE). Rooted in the concepts of incidental language learning (Saffran et al., 1997), learner autonomy (Little, 2007), and informal language learning (Bahrani and Sim, 2012), IDLE has emerged as a subfield of CALL in the language learning domain. IDLE is characterized by self-directed, informal English language learning that leverages a diverse range of digital devices, such as phones, computers, and laptops, as well as various resources like web applications and social media platforms, in informal settings (Zhang and Liu, 2022; Taherian et al., 2023). It is important to note that IDLE encompasses both form-focused activities, such as vocabulary acquisition applications, and meaning-focused tasks, such as engaging in English language discussions through YouTube video commenting, outside the formal classroom environment (Liu et al., 2023). As IDLE gains prominence, it offers a promising avenue for learners to engage with English language content and resources in informal contexts, augmenting their language learning experiences and potentially contributing to their overall language proficiency development.

Albeit the significant role of IDLE in shaping EFL learners’ affective variables (Lee and Drajati, 2019; Meng and Li, 2023), and influencing their performance (Mohammed Saeed Mohammed and Kaid Mohammed Ali, 2021; Anggraini et al., 2022) extant work is limited by a dearth of clarity about how this concept can contribute to student engagement in the context of EFL. In fact, to the best of our knowledge, there is no other study that investigates the correlation between IDLE and student engagement. This being said, regarding the association between IDLE and social presence, we are only aware of one study that attends this matter. Sun et al. (2017) examined the role of an IDLE tool (i.e., GRE Analytical Writing Section Discussion Forum) in affecting social presence of language learners. Their results indicated that IDLE could significantly contribute to the social presence of participants. Taken together, against this research gap, we tend to investigate the role of IDLE in predicting online learning engagement via the mediation of social presence in the context of EFL.

### The conceptual model

**Figure 1:**
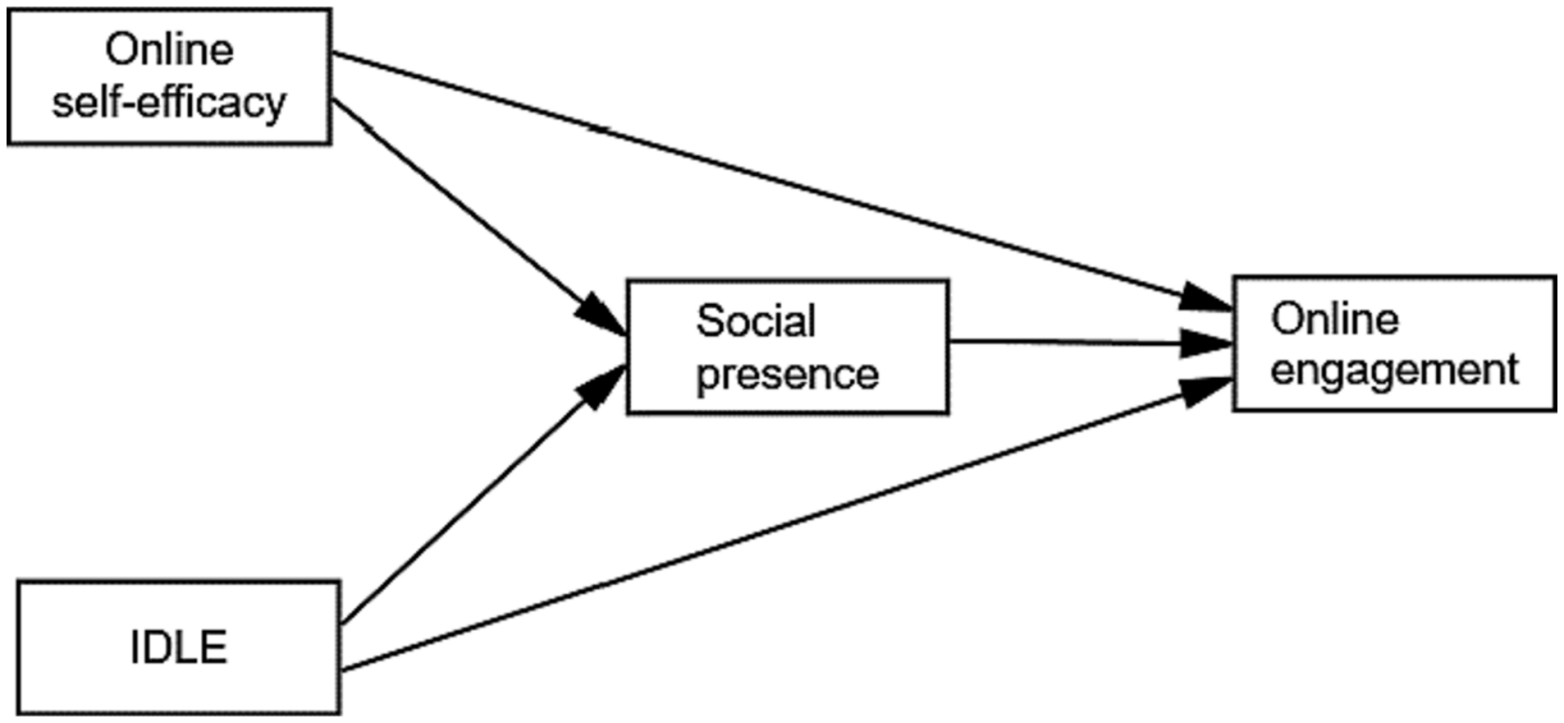
The conceptual model.

The proposed conceptual framework (see Figure 1) aims to explore the intricate relationships between online learning self-efficacy (OLSE), IDLE, social presence, and student engagement in online classes among intermediate EFL students. Each of these factors is hypothesized to play a crucial role in shaping students’ level of engagement in the virtual learning environment.

*H1*: OLSE is positively related to social presence in online classes.

Research indicates that self-efficacy, including OLSE, significantly impacts online learning (Tsai et al., 2020). Students with higher OLSE are more confident in their online learning abilities (Chang et al., 2014; Hsu et al., 2022), which can lead to increased engagement and interaction with instructors and peers, thereby fostering social presence (Cui et al., 2013; Hsu et al., 2022). In essence, when students believe in their online learning abilities, they are more likely to project their authentic selves and engage with others, ultimately enhancing social presence (Aragon, 2003; Doo and Bonk, 2020; Kuo et al., 2021). Therefore, it is reasonable to expect a positive relationship between OLSE and social presence in online classes.

*H2*: IDLE is positively related to social presence in online classes.

The emerging field of IDLE focuses on informal language learning in digital settings (Lee, 2019; Lee and Xie, 2023). Engaging in IDLE activities, such as online discussions or using language learning apps, offers additional language exposure and interaction opportunities (Lee and Drajati, 2019). These experiences likely translate into higher levels of social presence in online classes as students become more comfortable with digital interactions and engage more actively with instructors and peers (Lee et al., 2023). These interactions may contribute to a sense of comfort and connection, key components of social presence (Garrison, 2007), thus making it plausible that IDLE is positively related to social presence in online classes.

*H3*: Social presence is positively related to student engagement in online classes.

Extensive research supports the idea that social presence enhances student engagement in online learning environments (Harvey et al., 2018). When students perceive a higher level of social presence, they tend to feel more connected to their instructors and peers, fostering a sense of belonging and satisfaction in their interactions (Cui et al., 2013). This sense of connection and comfort encourages active participation, meaningful discussions, and collaboration, key components of student engagement (Molinillo et al., 2018; Miao et al., 2022). These positive feelings contribute to greater engagement as students actively participate in discussions, collaborate with peers, and invest more effort in their online coursework (Cui et al., 2013; Grieve et al., 2016). Therefore, it is reasonable to assume a positive relationship between social presence and student engagement in online classes.

*H4*: Social Presence mediates the relationship between OLSE and student engagement in online classes.

Building on the previous hypotheses, it is reasonable to expect that social presence acts as a mediator between OLSE and student engagement. OLSE, reflecting students’ confidence in their online learning abilities, can lead to increased social presence, as confident students are more likely to engage actively in online interactions (Alemayehu and Chen, 2021). This increased engagement is likely to be channeled through the enhancement of social presence, as students with high OLSE may project their authentic selves more effectively and engage more meaningfully with peers and instructors (Hsu et al., 2022; Wang et al., 2022). Consequently, social presence is expected to mediate the relationship between OLSE and student engagement in online classes.

*H5*: Social presence mediates the relationship between IDLE and student engagement in online classes.

Similar to Hypothesis 4, we propose that social presence acts as a mediator between IDLE and student engagement. Engaging in IDLE activities increases students’ comfort and proficiency in digital interactions, potentially leading to higher social presence in online classes (Lee and Xie, 2023). As social presence grows, it is expected to positively influence student engagement, as learners feel more connected and comfortable in their online learning environment (Whiteside et al., 2023). This increased social presence is expected to contribute to higher student engagement, as learners who feel more socially connected tend to participate actively and engage more deeply in their coursework (Miao et al., 2022). Therefore, it is reasonable to propose that social presence mediates the relationship between IDLE and student engagement in online classes.

Overall, this hypothesized model offers a thorough understanding of the factors influencing student engagement in online learning environments. The interplay between OLSE, IDLE, and social presence contributes to students’ level of engagement in online classes, highlighting the significance of self-efficacy beliefs and social interactions in fostering active participation and successful learning outcomes in the virtual learning context.

## Methodology

### Participants

The study comprised a sample of 389 intermediate Chinese EFL students who were enrolled in online English courses within a well-established chain of language schools. Careful selection criteria were applied to ensure a more homogeneous sample in terms of age group and English proficiency level. The age range of the participants fell between 18 to 25 years, resulting in a focused and consistent age group for the study. Moreover, all students were classified as intermediate-level learners in English proficiency.

Regarding gender distribution, 56% of the participants were female, while 44% were male, indicating a relatively balanced representation of both genders in the sample. The educational backgrounds of the participants varied and included high school graduates, college students, and working professionals, all seeking to enhance their English language skills through online learning. To gain insights into participants’ prior experience with online learning, relevant information was collected. Approximately 80% of the participants reported having prior exposure to online courses, while the remaining 20% were relatively new to the online learning format. This data ensured that the majority of the participants had some level of familiarity with virtual learning, while still including a significant proportion of learners new to the online environment.

Participants’ technological competence was also assessed, with 72% reporting being highly proficient in utilizing digital tools and online platforms, while the remaining 28% possessed moderate to basic technological skills. This diverse range of technological competence provided a comprehensive view of participants’ digital readiness and its potential influence on their engagement in online learning.

Exploring the motivations behind participants’ enrollment in online English courses revealed various driving factors. The primary reasons cited by the participants included improving career prospects (38%), enhancing communication skills (29%), and pursuing further education opportunities abroad (22%). These motivations reflected the specific goals and aspirations of learners within this particular age and proficiency group and shed light on their underlying incentives for engaging in online language learning.

### Instruments

#### IDLE scale

The IDLE Scale (see Appendix A), originally developed by Lee and Drajati (2019), was adapted for this study to assess informal digital English learning of the participants. It comprises four subscales measuring receptive and productive activities: form-focused activities (FF; 3 items), game-based activities (GB; 2 items), receptive IDLE activities (RE; 4 items), and productive IDLE activities (PR; 4 items). Participants indicated their engagement frequency in these activities on a 5-point Likert-type scale, ranging from 1 (never) to 5 (very often – many times per day). A sample item is “I use Google to check grammar and vocabulary.”

#### Online learning self-efficacy scale

Tsai et al. (2020) developed and validated the OLSS, which comprises 25 items encompassing five distinct aspects (see Appendix B): (1) Self-efficacy in successfully completing an online course, (2) Self-efficacy in engaging socially with peers, (3) Self-efficacy in navigating Course Management System (CMS) tools, (4) Self-efficacy in interacting with online instructors, and (5) Self-efficacy in collaborating with classmates for academic purposes. Participants were required to indicate their level of agreement with each statement using a 5-point Likert scale, ranging from 1 (strongly disagree) to 5 (strongly agree).

#### Social presence scale

To gauge social presence, a 10-item survey was utilized, adapted from Richardson and Swan’s (2003) scale on social presence (see Appendix C). This questionnaire was originally based on Gunawardena and Zittle’s (1997) social presence measure, but was modified to specifically capture social interactions in a specific online setting, rather than the broader focus on online learning in the original scale. An illustrative statement from the survey is “I felt comfortable introducing myself in this course.” Participants indicated their level of agreement with each statement using a Likert-type scale ranging from 1 (strongly disagree) to 6 (strongly agree).

#### Online student engagement scale

In this study, an assessment tool consisting of 16 items was employed to gauge online student engagement (see Appendix D), which was previously developed by Hoi and Le Hang (2021). The questionnaire utilized a five-point Likert scale and encompassed four distinct subscales: Behavioral engagement (BE), Cognitive engagement (CE), Affective engagement (AE), and Social engagement (SE). Each subscale was measured by four items. In particular, the scale measured Behavioral engagement (BE) to assess students’ active involvement and positive conduct, Cognitive engagement (CE) to evaluate their cognitive dedication, Affective engagement (AE) to gauge emotional responses, and Social engagement (SE) to measure efforts in maintaining relationships with peers and instructors.

### Procedure

Data collection for this research study followed a structured and standardized process, incorporating various methods to comprehensively assess the study variables. Participants were recruited from diverse language schools situated within a city known for its diverse student population and high demand for English language education. These language schools were part of a well-established chain of institutions offering online English courses, providing a suitable pool of participants for the study.

To ensure informed consent and voluntary participation, potential participants received detailed information about the study’s objectives, procedures, and the option to withdraw at any point without consequences. Written informed consent was obtained from all participants before their inclusion in the study, guaranteeing their voluntary involvement. Participants were then requested to complete a demographic questionnaire, gathering essential information such as age, gender, educational background, and English language proficiency level. Additionally, data on their prior experience with online learning, technological competence, socioeconomic background, motivations for enrolling in online English courses, time spent on online activities, and frequency of social interactions within the online learning platform were also collected. This comprehensive approach allowed for a thorough understanding of the participant characteristics and context.

To assess the study variables, participants were asked to complete four standardized scales of the constructs of interest. Data collection was facilitated through online platforms, allowing participants to conveniently complete the questionnaires and scales using their preferred devices, at their preferred time and location. This approach offered flexibility and accessibility, enhancing response rates and data quality. The data collection process spanned a period of 4 weeks, during which diligent efforts were made to maximize participant engagement and response rates. Frequent reminders and follow-ups were conducted to encourage participation and ensure representative data. Throughout the data collection process, strict confidentiality and anonymity of participants were maintained. All data were anonymized and stored securely, with access restricted to the research team to safeguard participant privacy.

### Data analysis

The gathered data underwent an initial analysis using SPSS version 28.0. Subsequently, correlation analyses were performed to investigate the connections between the variables of interest. To evaluate the research hypothesis, Structural Equation Modeling (SEM) was applied utilizing the Amos program (version 26.0). The first step involved fitting the measurement model to the data, following the two-step approach recommended by Kline (2023). During this stage, the construct validity of the measurement model was assessed by examining factor loadings, composite reliability, and average variance extracted (AVE) of the latent variables.

Subsequently, the structural model was examined to evaluate the interrelationships among the latent variables. The model’s fit was assessed using various fit indices. The χ^2^/df ratio was employed to determine the model’s goodness-of-fit, with a value of *p* greater than 0.05 indicating an acceptable fit (Hu and Bentler, 1999). Additional fit indices, such as the Goodness of Fit Index (GFI) and the Comparative Fit Index (CFI), were considered satisfactory if their values reached 0.90 or higher (Marsh et al., 2004). Additionally, the Root-Mean-Square Error of Approximation (RMSEA) and the Standardized Root-Mean-Square Residual (SRMR) were utilized to evaluate model fit, with RMSEA values <0.08 and SRMR values <0.10 indicative of a good fit (Hu and Bentler, 1999).

## Results

Initially, to assess the construct validity of the measurement models, confirmatory factor analysis (CFA) was conducted. Items with factor loadings less than 0.5 were removed from the analysis. Consequently, two IDLE items (ID9 and ID12), four OLSE items (OL4, OL10, OL18, and OL22), one SP item (SP5), and two OSE items (OS8 and OS19) were removed. Table 1 displays the remaining items along with their respective factor loadings for each scale. The final modified model fit the data well, with χ^2^ = 850.120, df = 535, *p* < 0.001, CFI = 0.970, GFI = 0.892, RMSEA = 0.031, SRMR = 0.048.

**Table 1:** Results of CFA.

Construct	Indicators	Factor loading (λ)	Standard error	Critical ratio (CR)
IDLE	ID1	0.72	0.05	14.40
	ID2	0.87	0.04	21.75
	ID3	0.78	0.05	15.60
	ID4	0.84	0.04	21.00
	ID5	0.75	0.06	12.50
	ID6	0.68	0.06	11.33
	ID7	0.79	0.05	15.80
	ID8	0.89	0.04	22.25
	ID10	0.71	0.06	11.83
	ID11	0.82	0.05	16.40
	ID13	0.74	0.06	12.33
OLSE	OL1	0.88	0.04	22.00
	OL2	0.89	0.04	22.25
	OL3	0.91	0.03	30.33
	OL5	0.87	0.04	21.75
	OL6	0.85	0.04	21.25
	OL7	0.83	0.05	16.60
	OL8	0.88	0.04	22.00
	OL9	0.90	0.03	31.00
	OL11	0.84	0.04	21.00
	OL12	0.79	0.04	21.50
	OL13	0.89	0.04	22.25
	OL14	0.83	0.05	16.60
	OL15	0.92	0.03	30.67
	OL16	0.86	0.04	21.50
	OL17	0.88	0.04	22.00
	OL19	0.85	0.04	21.25
	OL20	0.83	0.05	16.60
	OL21	0.89	0.04	22.25
	OL23	0.88	0.04	22.00
	OL24	0.88	0.04	22.00
	OL25	0.89	0.04	22.25
Social presence	SP1	0.87	0.04	21.75
	SP2	0.82	0.05	16.40
	SP3	0.83	0.05	16.60
	SP4	0.86	0.04	21.50
	SP6	0.88	0.04	22.00
	SP7	0.90	0.03	30.00
	SP8	0.87	0.04	21.75
	SP9	0.89	0.04	22.25
	SP10	0.84	0.04	22.00
OSE	OS1	0.88	0.04	22.00
	OS2	0.89	0.04	22.25
	OS3	0.87	0.04	21.75
	OS4	0.84	0.04	21.00
	OS5	0.88	0.04	22.00
	OS6	0.89	0.04	22.25
	OS7	0.87	0.04	21.75
	OS9	0.86	0.04	21.50
	OS10	0.89	0.03	29.00
	OS11	0.88	0.04	22.00
	OS12	0.89	0.04	23.00
	OS13	0.82	0.04	19.00
	OS14	0.75	0.06	12.50
	OS15	0.69	0.06	11.66
	OS16	0.79	0.05	15.80
	OS17	0.89	0.04	22.25
	OS18	0.71	0.06	11.83
	OS20	0.83	0.05	16.55
	OS21	0.74	0.06	12.66
	OS22	0.81	0.05	16.80

Then the study variables were subjected to descriptive analysis and correlation to examine their characteristics. Table 2 presents the mean scores and standard deviations for each construct. The participants reported an average score of 3.62 (SD = 0.69) on the IDLE scale, indicating a moderate level of engagement in informal digital learning activities related to English. OSE demonstrated a mean score of 2.97 (SD = 0.76), indicating a somewhat lower level of self-efficacy in online learning among the participants. Social Presence had the highest mean score of 3.83 (SD = 0.81), indicating a relatively strong sense of social presence experienced in the virtual learning environment. Lastly, the mean score for online learning engagement was 3.74 (SD = 0.77), indicating a relatively high level of active participation and engagement in online classes.

**Table 2:** Descriptive analysis.

	Mean	*SD*	Croanbach’s α	1	2	3	4
1. IDLE	3.62	0.69	0.812	1			
2. OSE	2.97	0.76	0.833	0.322**	1		
3. SP	3.83	0.81	0.796	0.396**	0.401**	1	
4. Engagement	3.74	0.77	0.803	0.425**	0.328**	0.542**	1

OSE, online learning self-efficacy; SP, social presence; Engagement, online learning engagement, **p < 0.01.

Reliability analysis was performed to assess the internal consistency of the constructs, and Cronbach’s α coefficients were calculated. The results revealed acceptable levels of internal consistency, with Cronbach’s α coefficients of 0.812 for IDLE, 0.833 for OSE, 0.796 for SP, and 0.803 for online engagement. These coefficients indicate that the items within each construct are reliable measures, and the constructs exhibit good internal reliability.

The correlations between the constructs were also examined. As shown in Table 2, OSE showed significant positive correlations with online engagement (*r* = 0.322, *p* < 0.01) and SP (*r* = 0.396, *p* < 0.01). Similarly, SP exhibited significant positive correlations with both engagement (*r* = 0.401, *p* < 0.01) and OSE (*r* = 0.315, *p* < 0.01). Notably, the strongest correlation was observed between SP and online engagement (*r* = 0.542, *p* < 0.01), indicating a robust association between students’ perceived social presence and their level of online learning engagement.

Following Fornell and Larcker (1981), we investigated convergent validity by examining the factor loadings, composite reliability (CR), and average variance extracted (AVE) for each construct (see Table 3). For all four constructs, the factor loadings were above the recommended threshold of 0.70, indicating good convergent validity as the items had a strong relationship with their respective constructs. Additionally, the CR values were all above 0.70, suggesting high internal consistency and reliability of the items within each construct. Furthermore, the AVE values for all constructs were above 0.50, meeting the criterion for convergent validity. This indicates that more than 50% of the variance in the items is attributed to their underlying constructs, demonstrating that the items in each construct are converging on a common latent variable.

**Table 3:** Convergent validity.

Construct	Factor loading (λ)	Composite reliability (CR)	Average variance extracted (AVE)
IDLE	0.81	0.812	0.611
OSE	0.83	0.833	0.676
SP	0.80	0.796	0.645
Engagement	0.86	0.803	0.693

OSE, Online Learning Self-Efficacy; SP, Social Presence.

Also, based on Straub et al. (2004), discriminant validity was assessed by comparing the square root of the AVE for each construct with the correlations between the constructs. The square root of the AVE represents the amount of variance in each construct that is unique and not shared with other constructs.

As indicated in Table 4, it is evident that the correlations between each pair of constructs are all lower than the corresponding square roots of AVE. This indicates good discriminant validity as the correlations are less than the variance explained by each construct, suggesting that the constructs are distinct and not highly correlated with each other. For example, the square root of the AVE for IDLE is 0.781, and its correlations with OSE, SP, and Engagement are 0.322, 0.396, and 0.425, respectively. All these correlations are lower than 0.781, indicating good discriminant validity for IDLE. Similarly, the square root of the AVE for OSE is 0.822, and its correlations with IDLE, SP, and engagement are 0.401, 0.396, and 0.328, respectively. Again, all these correlations are lower than 0.822, demonstrating good discriminant validity for OSE. The same pattern holds for SP and engagement, with their correlations being lower than their respective square roots of AVE, indicating good discriminant validity for both constructs.

**Table 4:** Discriminant validity.

	Square Root of AVE	Correlation with other constructs
IDLE	0.781	0.322
OSE	0.822	0.401
Social Presence	0.803	0.396, 0.328
Engagement	0.832	0.425, 0.542

AVE, Average Variance Extracted; IDLE, Informal Digital Learning of English; OSE, Online Learning Self-Efficacy; SP, Social Presence.

After confirming the adequate fit of the measurement model, we moved forward to evaluate alternative structural models to test our research hypotheses. Firstly, we compared the hypothesized partial mediation model (Model A) with the full mediation model (Model B), where all path coefficients from online self-efficacy and IDLE to online engagement were set to zero. Additionally, we explored a competing direct model (Model C), where all path coefficients to and from social presence were constrained to zero.

As seen in Table 5, Comparing the fit indices, it can be observed that Model A (Partial Mediation Model) had the best overall fit among the three models. Model A demonstrated the lowest χ^2^ value (1,067.334), indicating a closer match between the model and the observed data compared to Models B and C. Additionally, Model A showed higher GFI (0.854) and CFI (0.973) values, suggesting a better fit to the data than the other models. Furthermore, Model A exhibited the lowest RMSEA (0.036) and the highest TLI (0.961) values, indicating a smaller discrepancy between the model and the data and a better fit to the data, respectively. The SRMR value for Model A (0.055) was also the lowest among the three models, further supporting its superior fit.

**Table 5:** Results of fit indices of alternative models.

Model	χ^2^	df	Δχ^2^	GFI	CFI	RMSEA	TLI	SRMR
Direct Effect Model (C)	1245.678 **	622	–	0.812	0.902	0.065	0.894	0.178
Full Mediation Model (B)	1132.456 **	619	113.222	0.834	0.956	0.048	0.932	0.067
Partial Mediation Model (A)	1067.334 **	615	65.122	0.854	0.973	0.036	0.961	0.055

**Value of *p* < 0.001, df = degrees of freedom, Δχ^2^ = difference in chi-square values between the model and the subsequent model.

**Figure 2:**
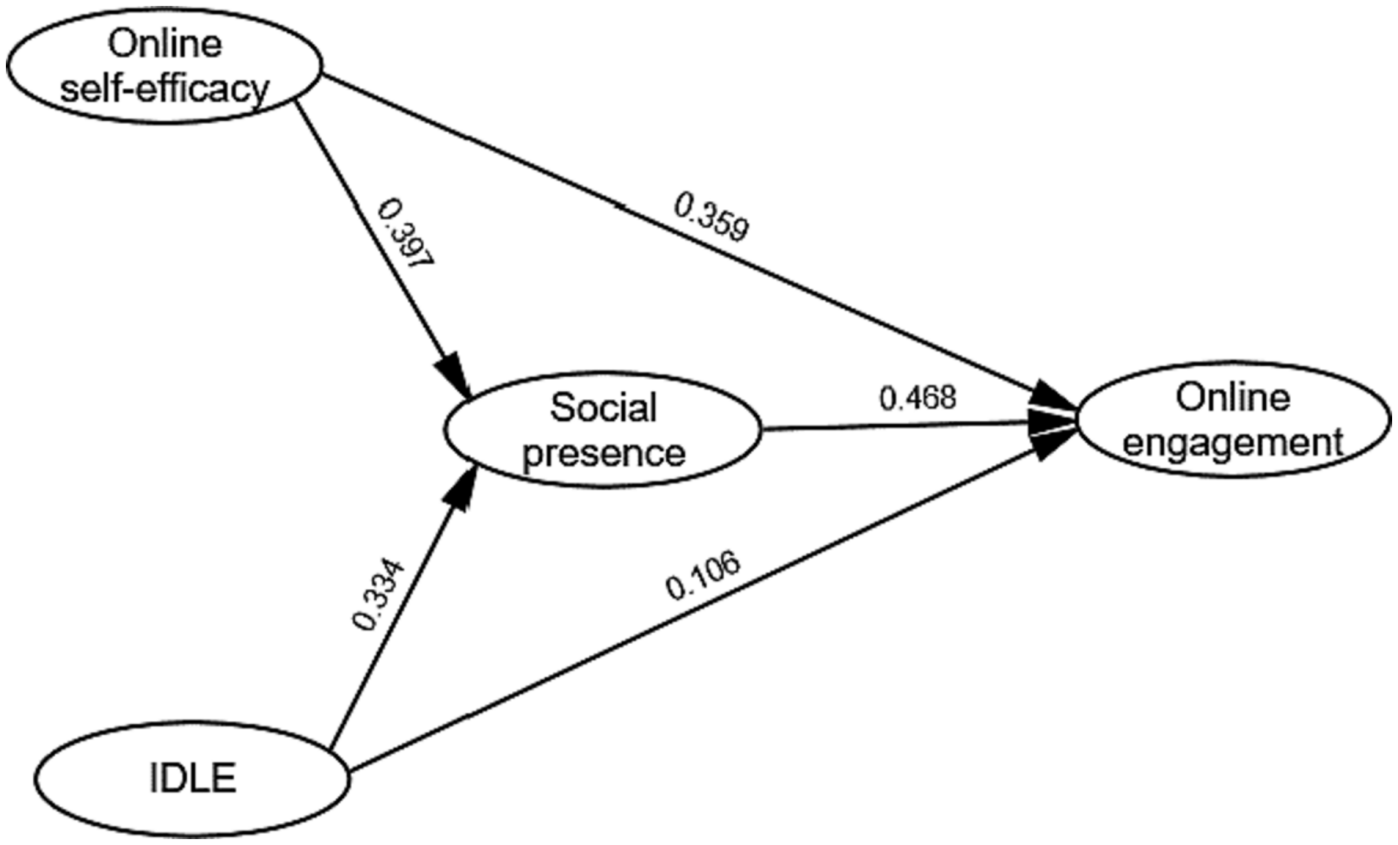
The final model.

Figure 2 illustrates the path and parameter estimates for the final partially mediated model (Model A). Most of the path coefficients were found to be statistically significant, except for the path connecting IDLE and online engagement, which was not statistically significant. To investigate whether social presence acted as a mediating variable among the study variables, we utilized the Baron and Kenny (1986) method (Table 6).

**Table 6:** Path estimates of structural model.

Standardized path coefficients (*t*-value)			
	Direct effects model	Full mediation model	Partial mediation model
OSE → Engagement	0.380 (5.02***)		0.359 (4.38**)
IDLE → Engagement	0.192 (2.76*)		0.106 (0.93)
OSE → SP		0.421 (5.37***)	0.397 (5.12***)
IDLE → SP		0.315 (3.87**)	0.334 (3.44**)
SP → Engagement		0.534 (7.23***)	0.468 (6.57***)

OSE, online learning self-efficacy; SP, social presence; Engagement, online learning engagement. *p < 0.05, **p < 0.01, ***p < 0.001.

In the direct effects model, the standardized path coefficient from OSE to online learning engagement was 0.380 (*t* = 5.02, *p* < 0.001), indicating a significant positive direct effect. The path from IDLE to online learning engagement was also positive, with a coefficient of 0.192 (*t* = 2.76, *p* < 0.05). The full mediation model constrained the paths from both OSE and IDLE to online engagement to zero. In this model, the path from OSE to social presence was 0.421 (*t* = 5.37, *p* < 0.001), while the path from IDLE to social presence was 0.315 (*t* = 3.87, *p* < 0.01). Additionally, the path from social presence to online engagement was 0.534 (*t* = 7.23, *p* < 0.001). In the partial mediation model, the path from OSE to online engagement was 0.359 (*t* = 4.38, *p* < 0.01), while the path from IDLE to online engagement was 0.106 (*t* = 0.93, not statistically significant). The paths from OSE to social presence (0.397, *t* = 5.12, *p* < 0.001) and from IDLE to social presence (0.334, *t* = 3.44, *p* < 0.001) were both significant. The path from social presence to online engagement was 0.468 (*t* = 6.57, *p* < 0.001).

The conditions for conducting mediation analysis were satisfied based on the findings, as all three criteria were met: (1) a significant association between the independent variables (online learning self-efficacy and IDLE) and the dependent variable (online engagement), (2) a significant association between the independent variables and the mediator (social presence), and (3) a significant association between the mediator and the dependent variable while considering the influence of the independent variables. The results of the mediation analysis demonstrated that social presence played a mediating role in the relationship between IDLE and online engagement in the model.

Lastly, to mitigate potential common method bias, a Harman’s single-factor test was performed, combining all latent variables assessed using self-reported measures. The outcome revealed that the first factor explained only 33.94% of the variance, falling below the 50% threshold. This result indicates the absence of substantial common method bias in the study.

## Discussion

The present research aimed to examine the predictive role of online learning self-efficacy, social presence, and IDLE on online learning engagement among EFL learners in online classes. The study holds significant implications for L2 learning and teaching in the digital context. The findings offer valuable insights into the complex dynamics that influence student engagement in the virtual learning environment and contribute to our understanding of the factors that promote active participation and successful learning outcomes in online education.

Our findings revealed a direct relationship between online learning self-efficacy and online learning engagement. This supports the existing body of literature highlighting the positive impact of online learning self-efficacy on various outcomes for L2 learners, including increased online learning engagement (Alemayehu and Chen, 2021; Han et al., 2021; Heo et al., 2021; Kuo et al., 2021; Wang et al., 2022). It can be inferred that higher levels of online self-efficacy may lead to heightened engagement among EFL students. Students who showed higher levels of OLSE were more engaged in their virtual coursework. This finding is in line with Bandura’s self-efficacy theory, which suggests that individuals who have confidence in their abilities are more likely to approach difficult tasks with greater effort and determination. In the context of online learning, students with robust OLSE may perceive themselves as more adept at navigating virtual platforms and handling the intricacies of online coursework. Consequently, they are more likely to demonstrate higher levels of engagement. This corroborates the observations made by Han and Wang (2021), who highlighted the pivotal role of learners’ self-efficacy beliefs in fostering engagement within the EFL context. Confidence in the online environment empowers learners to actively participate in digital tasks, thus enhancing their overall engagement. Furthermore, our findings align with those of Derakhshan and Fathi (2023), who also reported a positive predictive relationship between online self-efficacy and online engagement.

Furthermore, the results of our study indicated that social presence directly predicts online learning engagement. This finding aligns with previous research in the field of general education (e.g., Molinillo et al., 2018; Doo and Bonk, 2020; Miao et al., 2022), which has consistently demonstrated the significant predictive role of social presence in online learning engagement. The significance of social presence cannot be overstated in the context of online education. It functions as a linchpin, connecting learners with their social identity through interactions with peers and instructors, ultimately fostering their engagement in online learning activities. This finding aligns with the observations made by Doo and Bonk (2020), who emphasized that learners endowed with a heightened sense of social presence are better equipped to forge meaningful relationships and engage in effective communication within the online environment, thereby experiencing increased levels of engagement.

Moreover, our findings resonate with the principles of the Community of Inquiry (COI) framework, as expounded by Garrison et al. (1999). The COI framework underscores the interplay of three essential presences—social, cognitive, and teaching—in crafting a meaningful and effective online learning experience. In particular, social presence, as a fundamental dimension within the COI framework, assumes a pivotal role in constructing a supportive and interactive learning environment. When students perceive a heightened sense of social presence, they are more inclined to actively participate in collaborative learning activities, freely exchange ideas, and engage in substantive discussions. These behaviors collectively contribute to elevating their levels of engagement in the virtual classroom. Social presence, as a contributing factor, fosters an atmosphere where students feel socially present and acknowledged. This recognition, in turn, propels them to embrace collaborative learning and engage more deeply with the learning material.

Thirdly, our findings revealed that IDLE plays a significant predictive role in shaping learners’ online learning engagement, and this relationship is mediated by social presence. Our findings align harmoniously with previous research, particularly the work of Sun et al. (2017), which similarly highlighted the positive influence of IDLE on students’ social presence. In essence, the use of IDLE tools and applications contributes substantially to the development of learners’ social and communication skills (Anggraini et al., 2022), consequently fostering an augmented sense of social presence within the online learning environment. IDLE encompasses a rich tapestry of self-directed language learning activities that unfurl in digital settings. These activities may encompass the utilization of language-focused applications, consumption of language-related videos, or active participation in language interactions across various social media platforms—all conducted in the target language. These informal language learning experiences extend beyond the confines of the formal virtual classroom (Lee and Dressman, 2018), offering learners invaluable opportunities to immerse themselves in the language.

As a natural consequence, learners who enthusiastically engage in IDLE activities tend to experience heightened language exposure and engage in more extensive language practice, ultimately contributing positively to their overall language development (Lee, 2019). It is within this enriched linguistic environment that the nexus between IDLE and online learning engagement comes into sharper focus. Enhanced language proficiency, resulting from active involvement in IDLE endeavors, exerts a direct influence on learners’ online learning engagement. This influence stems from the enriched language skills acquired during IDLE activities, which empower learners to engage more fluently and confidently in online learning experiences within virtual L2 classrooms.

From this vantage point, it becomes evident that IDLE activities, particularly within the context of L2 learning, serve a dual purpose. On one hand, they enhance EFL learners’ social presence and communicative abilities, enabling them to traverse virtual learning landscapes with increased ease and confidence. On the other hand, this heightened sense of social presence within online learning environments catalyzes a ripple effect, positively impacting EFL learners’ online engagement. The resultant synergy fosters a higher degree of engagement in their online learning experiences.

Overall, our study has shed light on the intricate web of relationships between OLSE, IDLE, social presence, and student engagement in the context of online language learning. These findings contribute to a deeper understanding of the multifaceted dynamics at play within the virtual learning environment. Notably, OLSE emerges as a potent predictor of student engagement, echoing the importance of fostering learners’ confidence in navigating online platforms. Furthermore, IDLE serves as a catalyst for both enhanced language proficiency and social presence, offering learners an avenue to thrive in online language learning experiences. The mediating role of social presence underscores the significance of creating a vibrant online community that supports engagement. In essence, our study underscores the dynamic interplay between self-efficacy, informal digital learning, social presence, and engagement, paving the way for more informed strategies in online language education.

The current research has both theoretical and pedagogical implications. This study might be conducive to a better awareness and understanding of the nature, features, dimensionality, and practicality of online environment within SLA domain. In fact, it may enrich existing literature on association between/among online learning self-efficacy, social presence, IDLE and online learning engagement *via* delving into the indirect influence of IDLE on student engagement in virtual context through social presence. Furthermore, our study further may draw a fine-grained picture of how social presence and online learning self-efficacy contribute to online learning engagement. More importantly, the results of this study are significant to EFL administrators and development researchers interested in promoting the engagement of L2 students in online contexts. This being said, since online learning self-efficacy can greatly add values to EFL learners’ online engagement, it is of critical importance that EFL administrators and policy makers try to conduct and implement online classrooms and platforms which help students develop self-confidence and self-efficacy judgments in virtual contexts. By providing support, resources, and training to help students develop their online learning skills, educators can boost their confidence and competence in navigating virtual platforms and interacting with online coursework. Strengthening students’ self-efficacy can lead to increased motivation and active participation in online classes.

In addition, the study found that taking part in IDLE activities had a positive impact on student engagement in online classes. By incorporating informal language learning activities, such as language-focused online games and watching English videos, students had the opportunity to enhance their language skills beyond traditional classroom instruction. These IDLE activities provided valuable language exposure and practice, which complemented formal language learning and enriched the overall learning experience. As a result, students felt more connected to the language and became more actively engaged in their online coursework.

In addition, the social presence experienced by EFL students deserves further attention by course designers, researchers, and scholars, specifically in light of learners’ participation and engagement in online environments (Molinillo et al., 2018; Miao et al., 2022). Teachers can enhance online courses by fostering social interactions and collaboration among students, as well as between students and instructors. The integration of multimedia elements, discussion forums, and collaborative activities in online courses can lead to a more dynamic and stimulating virtual learning environment. These course designs, which aim to foster a sense of community and connectedness among learners, hold the potential to substantially enhance student motivation and contribute to sustained engagement throughout the entire learning process.

## Conclusion

In this study, we investigated the relationships between online learning self-efficacy, IDLE, social presence, and student engagement in online classes among intermediate EFL students. Our results support the importance of online learning self-efficacy as a crucial predictor of student engagement in online classes. Higher levels of OLSE positively influenced students’ online learning engagement, confirming the significance of learners’ confidence in navigating and excelling in virtual learning environments. Social presence directly predicted student engagement in online classes Furthermore, social presence emerged as a key mediator in the relationship between IDLE and student engagement. As students engaged in informal digital language learning activities, they also developed a stronger sense of social presence within the virtual community, fostering connections with classmates and instructors. This heightened social presence positively influenced student engagement, as learners felt more connected and invested in their online learning experiences.

Although this study has provided valuable insights into the determinants of student engagement in online learning, it is essential to acknowledge certain limitations. First, the focus of this study was on a specific group of intermediate Chinese EFL students within a chain of language schools. Consequently, the generalizability of the findings to other student populations with different cultural backgrounds, educational levels, or language proficiencies may be limited. Future research should incorporate diverse samples from various contexts to examine the relationships among the study variables more comprehensively. Second, the study employed a cross-sectional design, which hinders the establishment of causal relationships between the study variables. To obtain more robust evidence of the causal effects and temporal dynamics among online learning self-efficacy, IDLE, social presence, and student engagement, longitudinal studies or experimental designs should be considered.

Third, the data were collected through self-report measures, which might introduce response bias and social desirability effects. To enhance the validity and reliability of the findings, future research could incorporate multiple data sources, such as objective assessments of student engagement or observations of online interactions. Lastly, the study did not explicitly examine certain contextual factors that could influence student engagement in online classes, such as the design of specific online courses, the level of instructor support, or the technological resources available to students. Incorporating these contextual variables in future research endeavors would lead to a more comprehensive understanding of the factors influencing student engagement in online education.

## Data availability statement

The raw data supporting the conclusions of this article will be made available by the authors, without undue reservation. Requests to access these datasets should be directed to RW, zjsruwrl@163.com.

## Ethics statement

The studies involving humans were approved by School of Humanity and Foreign Languages, Zhejiang Shuren University. The studies were conducted in accordance with the local legislation and institutional requirements. The participants provided their written informed consent to participate in this study.

## Author contributions

RW: Conceptualization, Data curation, Formal analysis, Funding acquisition, Investigation, Methodology, Project administration, Resources, Software, Supervision, Validation, Visualization, Writing – original draft, Writing – review & editing.
