# Supplementary material for: The relationship between online learning self-efficacy, informal digital learning of English, and student engagement in online classes: the mediating role of social presence
Source: Front Psychol. 2023 Oct 2;14:1266009. doi: 10.3389/fpsyg.2023.1266009 (PMC10577387; doi:10.3389/fpsyg.2023.1266009)
Supplement: Supplementary file 1 [file Data_Sheet_1.docx]

**Appendices**

***Appendix A:* IDLE Scale**

*Receptive IDLE activities*

1. I play games in English.

2. I listen to English language news programmes online or on TV.

3. I listen to songs in English.

4. I listen to English podcasts.

5. I watch English comics online or on TV.

6. I watch sports events in English online or on TV.

7. I watch English language movies or dramas with subtitles in English.

*Productive IDLE activities*

1. I chat with others in English via social media (e.g. Facebook, KaKaoTalk, Line, WeChat, WhatsApp).

2. I Skype with others in English.

3. I send an email to others in English.

4. I share English contents online.

5. I use technology to connect with native speakers of the language (e.g. British, American).

6. I use technology to connect with non-native speakers of English all over the world (e.g. Japanese, Chinese).

***Appendix B:* OLSS**

*Factor 1: Self-efficacy to complete an online course*

1. Willing to face challenges
2. Create a plan to complete the given assignments
3. Willingly adapt my learning styles to meet course expectations
4. Understand complex concepts
5. Keep up with course schedule
6. Evaluate assignments according to the criteria provided by the instructor
7. Complete an online course with a good grade

*Factor 2: Self-efficacy to interact socially with classmates*

1. Pay attention to other students’ social actions
2. Initiate social interaction with classmates
3. Apply different social interaction skills depending on situations
4. Develop friendship with my classmates

*Factor 3: Self-efficacy to handle tools in a CMS*

1. Send email to others with or without attached files
2. Reply to others’ messages in a discussion board
3. Post a new message in a discussion board

*Factor 4: Self-efficacy to interact with instructors in an online course*

1. Clearly ask my questions to instructor
2. Seek help from instructor when needed
3. Timely inform the instructor when unexpected situations arise
4. Initiate discussions with the instructor
5. Express my opinions to instructor respectfully

*Factor 5: Self-efficacy to interact with classmates for academic purposes*

1. Actively participate in online discussions
2. Effectively communicate with my classmates
3. Respond to other students in a timely manner
4. Request help from others when needed
5. Express my opinions to other students respectfully
6. Provide help to other students when assistance is needed

***Appendix C:* Social Presence Scale**

1. Getting to know other course participants gave me a sense of belonging in the course
2. I was able to form distinct impressions of some course participants
3. Online or web-based communication is an excellent medium for social interaction
4. I felt comfortable conversing through the online medium
5. I felt comfortable participating in the course discussions
6. I felt comfortable interacting with other course participants
7. I felt comfortable disagreeing with other course participants while still maintaining a sense of trust
8. I felt comfortable introducing myself in this course
9. I felt that my point of view was acknowledged by other course participants
10. Online discussions help me to develop a sense of collaboration.

***Appendix D:* Online Student Engagement Scale**

*Behavioural engagement*

1. I take notes when I participate in online discussions.
2. I stay focused during online learning activities.
3. I talk about online learning topics even when I am offline.
4. I complete all online learning tasks on time.

*Cognitive engagement*

1. I go through learning materials before I participate in online discussions.
2. I try to connect what I am learning online with what I learn before.
3. I try to find extra learning resources to understand a difficult concept when learning online.
4. I try to understand my mistakes if I get something wrong during online learning activities.

*Affective engagement*

1. I enjoy online learning activities.
2. I look forward to online learning activities.
3. I feel comfortable participating in online discussions.
4. I feel inspired to improve my online learning skills.

*Social engagement*

1. I share learning materials with other online classmates.
2. I build on other ideas during online discussions.
3. I ask teachers if I do not understand something when learning online.
4. I respond to other classmates' questions in online discussion boards.
